# Sentinel node biopsy and radical lymph node dissection for advanced melanoma in the elderly

**DOI:** 10.1186/1471-2318-11-S1-A9

**Published:** 2011-08-24

**Authors:** V Desiato, S Perrotta, GL Benassai, G Quarto, G Benassai, G Limite

**Affiliations:** 1 Dipartimento Universitario di Chirurgia Generale, Geriatrica, Oncologica e Tecnologie Avanzate Facoltà di Medicina e Chirurgia, Università degli Studi di Napoli Federico II, Naples, Italy

## Background

The majority of indications for surgery in melanoma are for the treatment of primary tumor and lymph node metastases. During the last decade, the Sentinel Node Biopsy (SNB), from a research procedure, has become standard of care in most institutions. SNB is normally considered for patients with melanoma > 1 mm and generally about 20% are positive; however, the risk of a positive SNB in a melanoma < 1 mm is still 5%. Usually when SNB is positive a complete lymphadenectomy is performed.

## Materials and methods

In the period 2004-2009, 18 elderly patients (median age 68 years) affected by cutaneous melanoma (mean Breslow’s thickness = 3.77 mm), after SNB histologically confirmed regional lymph node involvement, underwent complete lymph node dissection (CLND). We treated 11 of them with groin dissection, in 3 cases bilateral; 4 patients underwent axillar dissection, in one case bilateral; 2 patients underwent neck dissection and another patient underwent groin-axillar dissection. We treated bilateral groin involvement with laparoscopic access for dissection of lumbar-aortic, iliac and obturator lymph nodes.

## Results

Disagreeing with literature, 12/18 (67%) of these patients had positive lymph nodes, a high percentage if compared with younger patients’ data. Currently the average follow-up is 25 months. In our sample CLND has a crucial prognostic role (16% vs 41% of deceased in CLND – and CLND + patients respectively).

## Conclusions

Elderly melanoma patients are characterized by a higher tumor stage and, in patients with nodal metastases, the prognosis is independently affected by older age. In case of positive SNB the CLND plays a notable prognostic role and a presumable therapeutic role.

## References

[B1] Marsden JR Newton-Bishop JA Revised U.K. guidelines for the management of cutaneous melanoma 2010 Br J Dermatol 2010 163 2 238 56 10.1111/j.1365-2133.2010.09883.x 20608932

[B2] Testori A Stanganelli I Diagnosis of melanoma in the elderly and surgical implications Surg Oncol 2004 13 4 211 21 10.1016/j.suronc.2004.09.002 15615659

[B3] Kruijff S Bastiaannet E Detection of Melanoma Nodal Metastases; Differences in Detection Between Elderly and Younger Patients Do Not Affect Survival Ann Surg Oncol 2010 17 3008 3014 10.1245/s10434-010-1085-1 20443146 PMC2950925

